# Primary peri-implant oral intra-epithelial neoplasia/carcinoma in situ: a case report considering risk factors for carcinogenesis

**DOI:** 10.1186/s40729-017-0109-z

**Published:** 2017-11-16

**Authors:** Makoto Noguchi, Hiroaki Tsuno, Risa Ishizaka, Kumiko Fujiwara, Shuichi Imaue, Kei Tomihara, Takashi Minamisaka

**Affiliations:** 1 Department of Oral and Maxillofacial Surgery, Graduate School of Medicine and Pharmaceutical Sciences for Research, University of Toyama, 2630 Sugitani Toyama city, Toyama, 9300194 Japan; 2 Department of Diagnosis Pathology, Graduate School of Medicine and Pharmaceutical Sciences for Research, University of Toyama, Toyama, Japan

**Keywords:** Dental implant, Oral intra-epithelial neoplasia/carcinoma in situ, Peri-implantitis, Risk factor for oral carcinogenesis

## Abstract

**Background:**

Major risk factors for oral squamous cell carcinoma (SCC) are tobacco smoking, a betel quid chewing habit, and heavy alcohol consumption. However, around 15% of oral SCCs cannot be explained by these risk factors. Although oral SCC associated with dental implants is quite rare, there has been a recent gradual accumulation of reports about it. Here, we report a case of primary peri-implant oral intra-epithelial neoplasia/carcinoma in situ (OIN/CIS) in a woman without the major risk factors for oral SCC.

**Case presentation:**

A 65-year-old woman was referred to our clinic with a tumor in the right lower gingiva. She had no history of tobacco smoking and only drank socially. Ten years previously, mandibular right posterior teeth had been replaced with an implant-supported porcelain-fused-to-metal restoration in a dental clinic. About 7 years later, she noticed swelling on the lingual side of the gingiva around the implant-supported restoration, and was eventually referred to our clinic with the suspicion of a neoplasia around the dental implant. The upper part of the implant body was exposed on the implant corresponding to the first molar of the right side of the mandible; this was associated with painless, elastic soft, and relatively well circumscribed gingival swelling on the lingual site. A panoramic radiograph showed slight vertical bone resorption around the implants. An incisional biopsy was conducted under the suspicion of neoplasia. Pathological microscopic examination of the biopsy specimen revealed thickened squamous epithelia with slight nuclear atypism and disorders of the epithelial rete pegs. Immunohistochemical findings showed positive staining for keratin 17 and a negative staining mosaic pattern for keratin 13. High p53, p63, and Ki-67 reactivity was also observed. From these findings, OIN/CIS of the gingiva was pathologically diagnosed, and a wide local excision with rim resection of the mandible, including the implants, was performed. The pathological findings for the resected specimen were same as those for the biopsy specimen. After 1 year of follow-up, there was no evidence of recurrence.

**Conclusion:**

In this case, prolonged peri-implant mucositis or peri-implantitis may have been a plausible risk factor for carcinogenesis.

## Background

Oral cancer ranks sixth among the malignancies in terms of worldwide prevalence, with more than 90% being pathologically squamous cell carcinoma (SCC) [1]. Oral SCC generally develops via multistep carcinogenesis. The squamous epithelium goes into irreversible change, including epithelial dysplasia and oral intra-epithelial neoplasia/carcinoma in-situ (OIN/CIS) [2], finally resulting in the development of invasive carcinoma through the accumulation of genetic abnormalities caused by persistent exposure to a carcinogen. Risk factors for oral SCCs are smoking, tobacco and betel quid chewing habits, and heavy alcohol consumption. In addition, chronic inflammation, including periodontitis, has been regarded as a possible risk factor for oral SCCs. Laprise et al. [3] conducted a case control study to estimate the extent to which high levels of periodontal disease were associated with oral cancer risk, using a comprehensive adjustment approach for confounding that involved a large set of life course variables. The authors concluded that their findings supported the hypothesis that high levels of periodontal disease increase the risk of oral cancer.

A recent report made a bold statement that dental implants also can be a cause of cancer by providing a “route of entry for squamous cell carcinoma” [4], and another report stated that dental implants can lead to SCC in at-risk patients [5]. The incidence of malignances associated with dental implants seems to be extremely low. Bhatavadekar [6] calculated the theoretical standardized incidence ratio (SIR) to be 0.0017 per million per year. However, recently, case reports of oral malignancies associated with dental implants have gradually been accumulating [7]. Raise et al. [8] pointed out that the number of such cases in the literature has increased sharply in the last decade.

In this study, we report a case of intra-epithelial neoplasia arising from peri-implant mucosa in a woman without a history of tobacco smoking or excessive alcohol consumption, and without predisposing factors for oral SCC, including leukoplakia, erythroplakia, or previous oral cancer.

## Case presentation

A 65-year-old woman was referred to our clinic with a tumor in the right lower gingiva. Her medical history included breast cancer without metastatic lesion, diabetes mellitus, hyperlipidemia, and hypertension. She had taken orally aspirin, amlodipine, pravastatin, and bepotastine for 2 years. She drank alcohol socially, but she had no history of tobacco smoking habit.

About 10 years prior to her attendance at our clinic, her mandibular right posterior teeth had been replaced with an implant-supported porcelain-fused-to-metal restoration (three endosseous hydroxyapatite-coated titanium implants) in a dental clinic. The postoperative clinical course was uneventful, and the implant-supported restoration had functioned well. The patient had discontinued her regular follow-up for the maintenance of oral hygiene several months after the completion of the restoration. After about 7 years, she noticed a swelling on the lingual side of the gingiva around the three implants, and she visited a dental clinic. Following conservative treatment with nonsurgical measures for around 2 years, under a diagnosis of peri-implantitis, flap surgery to her lesion was performed in the dental clinic. However, the lesion around the central of the three implants did not improve, and changes to its surface properties were observed, leading to the suspicion of transformation to neoplasia. She was then referred to our clinic 3 months after the flap surgery.

**Fig. 1:**
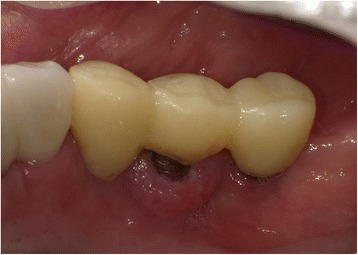
Well-circumscribed gingival swelling on the lingual side of the right side of the mandible

**Fig. 2:**
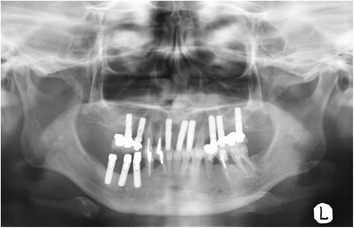
Panoramic radiograph shows slight vertical bone resorption around the implants in the right side of the mandible

**Fig. 3:**
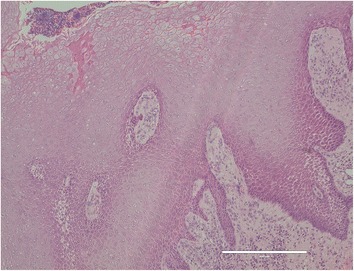
Pathological microscopic examination reveals thickened squamous epithelia with slight nuclear atypism and disorders of the epithelial rete pegs accompanied by moderate grade inflammatory cell infiltration (HE staining, bar: 400 μm)

**Fig. 4:**
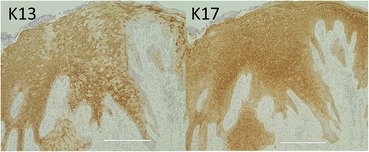
Immunohistological findings show a negative staining mosaic pattern for keratin 13 (k13) and positive staining for keratin 17 (k17) (immunohistological staining, bar 400 μm)

**Fig. 5:**
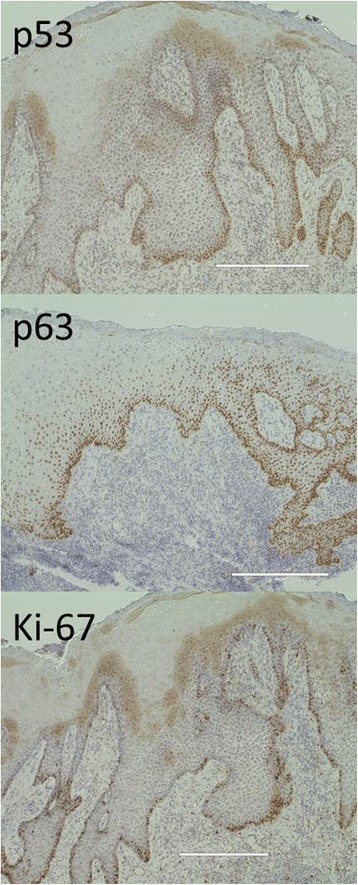
High p53, p63, and Ki-67 reactivity are also observed in the basal cell layer (immunohistological staining, bar 400 μm)

**Fig. 6:**
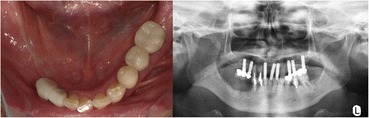
Postoperative intraoral finding and radiograph

The upper part of the implant body was exposed on the implant corresponding to the first molar of the right side of the mandible; this was associated with painless, elastic soft, and relatively well circumscribed gingival swelling on the lingual site. No pus drainage from the gingival sulcus was observed (Fig. 1). Periodontal disease or peri-implant disease, including peri-implantitis and peri-implant mucositis, were not observed in other regions, including the contiguous implants to the relevant middle implant. Lymphadenopathy in the neck was not detected. A panoramic radiograph showed slight vertical bone resorption around the three implants in the right side of the mandible (Fig. 2). An incisional biopsy was conducted under the suspicion of neoplasia after considering not only the clinical findings, but also the clinical course. Pathological microscopic examination of the biopsy specimen revealed thickened squamous epithelia with slight nuclear atypism and disorders of the epithelial rete pegs accompanied by moderate grade inflammatory cell infiltration. Immunohistochemical findings showed positive staining for keratin 17 (k17) and a negative staining mosaic pattern for keratin 13 (k13). High p53, p63, and Ki-67 reactivity was also observed in the basal cell layer, but negative staining for p16 (Table 1). These findings indicated to OIN/CIS. Thus, a wide local excision with rim resection of the mandible, including the three implants, was performed under general anesthesia. The postoperative clinical course was uneventful but the patient experienced paresthesia of the lower lip and mental region of the affected side. The pathological diagnosis of the resected specimen confirmed the OIN/CIS found in the biopsy specimen (Figs. 3, 4 and 5). The surgical margin was involved with epithelial dysplasia but free of OIN/CIS. After 1 year of follow-up, there was no evidence of recurrence (Fig. 6).

**Table 1:** Summary of immunohistochemical findings of the present case

Antibody	Sorce	Clone	Staining
Keratin 13	DAKO	DE-K13	Positive
Keratin 17	DAKO	E3	Negative mosaic pattern
p16	Roche	E6H4	Negative
p53	DAKO	DO-7	Positive in the basal cell layer
p63	Nichirei	4A4	Positive in the basal cell layer
Ki-67	Nichirei	SP6	Positive in the basal cell layer

### Discussion

OIN/CIS can sometimes be difficult to distinguish pathologically from epithelial dysplasia on hematoxylin- and eosin-staining sections; this has proved challenging for oral pathologists [9]. Recently, it has been reported that combined immunohistochemistry for k13 and k17 was useful for the differential diagnosis [9, 10]. K13 is a marker for cellular differentiation toward prickle cells in normal stratified squamous epithelia, and its loss or attenuation is observed in oral SCC. Conversely, k17 is not expressed in normal stratified squamous epithelia or specifically localized in SCCs. Our case showed a partial reduction in k13 expression and a mosaic-like pattern as well as being positive for k17 expression, leading to a diagnosis of OIN /CIS.

In the irreversible multistep carcinogenesis, most cases of invasive oral SCC that break through the basement membrane develop as the next pathological stage into OIN/CIS following severe epithelial dysplasia. Such precancerous lesions are clinically observed to be leukoplakic, erythroplakic, or a combination of these. Oral SCCs are frequently accompanied by such precancerous lesions which spread out around them.

Raiser et al. [8] reviewed 42 cases of oral malignancy in which dental implants were implicated, retrieved from a literature search of PubMed and Google Scholar. From the analysis, the affected individuals tend to be elderly adults (mean age, 68 years). The gender distribution shows a clear 1:1.5 female predominance opposed to the characteristic male predominance of oral cancer in general. They also found that 45.3% of cases occurred in a population with recognized risk factors for oral cancer; in addition, 47.5% of cases had experienced a previous oral malignancy, and 19% exhibited the histology of a non-oral malignancy that could metastasize to the jaws or gingiva. Our patient did not have any of the recognized risk factors for oral cancer, including a tobacco-smoking habit or heavy alcohol consumption, nor were further predisposing factors for oral cancer, such as a precancerous lesion or previous oral cancer observed in her oral cavity, including around the OIN/CIS lesion. Thus, she did not fit with the concept of “field cancerization” in the development of oral cancer, which has been accepted by most oral pathologists since being proposed in 1953 by Slaughter et al. [11].

The latest evidence implies that the human papilloma virus (HPV) may be responsible for carcinogenesis in the oral cavity [12, 13]; however, its role is debatable. The interaction of the HPV’s E6 and E7 oncoproteins with cell cycle proteins disturbs the cell cycle mechanism and subsequent alteration in the expression of proteins such as p53, p63, and Ki-67 [14]. In our case, the immunohistochemical examination of these cell cycle-related proteins demonstrated overexpression in the basal cell layer, which might be implicated in HPV infection and carcinogenesis. p16 has been regarded as one of the useful markers for HPV-associated carcinogenesis as well as other cell cycle proteins. Particularly in oropharyngeal SCCs, overexpression of p16 notably demonstrates a strong correlation with HPV infection, although it has failed to reveal such strong correlation with oral SCCs [15]. In our case, negative expression of p16 was observed, suggesting negative for HPV infection.

The relationship between the oral microbiome and oral SCC has increasingly been reported since the 1998 study by Nagy et al. [16], which found significantly higher levels of Porphyromonas species and Fusobacterium species on oral SCC, compared with the adjacent healthy mucosa. Recently, Gallimidi et al. [17] indicated that the periodontal pathogens *Porphyromonas gingivalis* and *Fusobacterium nucleatum* stimulated tumorigenesis via direct interaction with oral epithelial cells through toll-like receptors.

Evidence that persistent chronic inflammation may be a causative factor for carcinogenesis has accumulated from preclinical and clinical studies since Virchow proposed a close relationship between chronic inflammation and tumorigenesis [18]. It has been shown that chronic infection and related inflammation contributed to almost 20% of all malignancies worldwide. Various mechanisms for carcinogenesis related to chronic inflammation have been suggested. Inflammatory cells excrete a number of cytokines and growth factors that promote the survival of neoplastic cells and prevent their apoptosis [19]. Reactive oxygen and nitrogen species induced by chronic inflammation could cause damage to cellular deoxyribonucleic acid (DNA), contributing to malignant cell transformation [20]. Furthermore, a recent study suggested that inflammation could initiate cancer-specific epigenetic changes, including DNA methylation alterations in epithelial cells [21].

## Conclusions

In our case, the persistence of peri-implant mucositis or peri-implantitis around the dental implant was implicated as being a plausible risk factor for carcinogenesis. Regular follow-up to ensure the maintenance of oral hygiene after dental implant therapy has again been shown to be important for preventing peri-implantitis, a plausible risk factor for carcinogenesis.

## Abbreviations

DNA: Deoxyribonucleic acid
HPV: Human papilloma virus
OIN/CIS: Oral intra-epithelial neoplasia/carcinoma in situ
SCC: Squamous cell carcinoma
